# Effect of mechanical ventilation on intra-abdominal pressure in critically ill patients without other risk factors for abdominal hypertension: an observational multicenter epidemiological study

**DOI:** 10.1186/2110-5820-2-S1-S22

**Published:** 2012-12-20

**Authors:** Caridad de Dios Soler Morejón, Teddy Osmin Tamargo Barbeito

**Affiliations:** 1 Internal and Intensive Care Medicine, Intensive Care Unit, Hermanos Ameijeiras Hospital, San Lázaro and Belascoaín, La Habana, CP 10300, Cuba; 2 Bioestatistical Medicine, Department of Research and Development, Hermanos Ameijeiras Hospital, San Lázaro and Belascoaín, La Habana, CP 10300, Cuba

## Abstract

**Background:**

Mechanical ventilation (MV) is considered a predisposing factor for increased intra-abdominal pressure (IAP), especially when positive end-expiratory pressure (PEEP) is applied or in the presence of auto-PEEP. So far, no prospective data exists on the effect of MV on IAP. The study aims to look on the effects of MV on IAP in a group of critically ill patients with no other risk factors for intra-abdominal hypertension (IAH).

**Methods:**

An observational multicenter study was conducted on a total of 100 patients divided into two groups: 50 patients without MV and 50 patients with MV. All patients were admitted to the intensive care units of the Medical and Surgical Research Centre, the Carlos J. Finlay Hospital, the Julio Trigo University Hospital, and the Calixto García Hospital, in Havana, Cuba between July 2000 and December 2004. The IAP was measured twice daily on admission using a standard transurethral technique. IAH was considered if IAP was greater than 12 mmHg. Correlations were made between IAP and body mass index (BMI), diagnostic category, gender, age, and ventilatory parameters.

**Results:**

The mean IAP in patients on MV was 6.7 ± 4.1 mmHg and significantly higher than in patients without MV (3.6 ± 2.4 mmHg, *p *< 0.0001). This difference was maintained regardless of gender, age, BMI, and diagnosis. The use of MV and BMI were independent predictors for IAH for the whole population, while male gender, assisted ventilation mode, and the use of PEEP were independent factors associated with IAH in patients on MV.

**Conclusions:**

In this study, MV was identified as an independent predisposing factor for the development of IAH. Critically ill patients, which are on MV, present with higher IAP values on admission and should be monitored very closely, especially if PEEP is applied, even when they have no other apparent risk factors for IAH.

## Background

As stated in the consensus report from 2004, in the critically ill patient, intra-abdominal pressure (IAP) is frequently elevated above the patient's normal baseline IAP level which is approximately 5 to 7 mmHg in critically ill adults [1]. Many factors such as recent abdominal surgery, sepsis, organ failure, need for mechanical ventilation, and changes in body position have all been reported in association with elevations in IAP, and thus intra-abdominal hypertension (IAH) [1]. Mechanical ventilation (MV) can act as a predisposing factor for the elevation of the IAP [2-4], in particular, when it is associated with the use of positive end-expiratory pressure (PEEP) or in the presence of auto-PEEP.

On the other hand, the effects of IAP on the respiratory system have been well studied [5]. Increased IAP affects respiratory function with a profound impact on daily clinical practice [5]. The changes associated with elevated IAP include increased chest wall elastance (or thus decreased compliance), cranial shift of the diaphragm, with consequent reduction in the lung volume and atelectasis formation, lung edema, ventilator-induced lung injury, and reduced lymphatic flow in normal and impaired lungs [5].

The situation turns more complicated when the patient at risk for IAH is being mechanically ventilated because under analgosedation and/or muscle relaxation, the typical signs and symptoms of complications such as abscesses or intra-abdominal fluid collections, hematomas, or even diffuse peritonitis could be masked. These conditions, that can be very deleterious through the well-known effects of IAH on hemodynamics, respiratory and renal function, hepatosplanchnic perfusion and, therefore, for the whole body [2,6,7] could be aggravated by the use of MV *per se*.

The aim of the present study is to look on the effects of MV on baseline IAP values in critically ill patients. To the best of our knowledge, this has not been studied before. So far, no prospective data is available on the effect of MV on IAP although this situation is of special importance for intensive care unit (ICU) patients.

## Methods

### Patients

An observational multicenter and prospective study was conducted including a total of 100 critically ill patients without apparent risk factors for IAH other than the use of MV. The patients were admitted at the ICUs of the Medical and Surgical Research Centre, the Carlos J. Finlay Hospital, the Julio Trigo University Hospital, and the Calixto García Hospital between July 2000 and December 2004.

Patients of both genders were consecutively included according to feasibility criteria (the availability of resources for the measurement and the presence of the investigator in charge of the measurements). After their inclusion, the patients were separated into two groups: 50 critically ill patients were mechanically ventilated and 50 critically ill patients were not mechanically ventilated.

The criteria for the selection of the sample were established as follows:

Inclusion criteria include:

• patients with no abdominal medical or surgical pathology for the last 3 months or during the first 24 h before entry and

• patients that had a vesical catheter already in place, without signs of urinary tract infection or urologic sepsis.

Exclusion criteria (to avoid other factors related to IAH) were as follows:

• patients with abdominal surgery or with suspicion of a surgical abdomen, abdominal distension, ascites, present or recent pregnancy, and abdominal or pelvic trauma and

• patients who were administered a volume of fluids higher than 5 l of crystalloids or colloids in the preceding 24 h.

The recruited patients were distributed in each of the five pre-established age groups (<30, 31 to 40, 41 to 50, 51 to 60 and, finally, 60 years and older). In relation with the diagnosis, the patients were also separated into two major categories, medical and surgical; the latter was divided into elective, emergency (E), and trauma (T) patients. The indications for MV in the medical patients were categorized as follows: respiratory failure (RF), cerebrovascular problems with diminished mental status (GCS<8), acute myocardial infarction (AMI), metabolic disorders (MD), congestive heart failure (CHF), and others; in the surgical group, the elective patients corresponded to cardiovascular surgery (CVS) and neurosurgery (NS).

### Measurements

**Figure 1:**
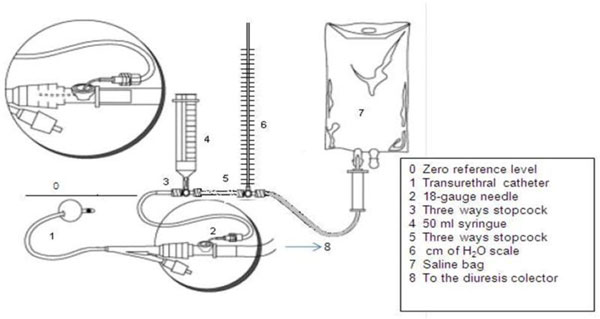
**IAP measurement technique**. A centimeter of water scale is inserted instead of a transducer. Adapted from Cheatham and Safcsak's technique [8] (reprinted with permission from the author). MV baseline settings were as follows: FiO_2 _0.5, tidal volume (Vt) 6 to 8 ml/kg, no PEEP, respiratory rate (RR) 16 to 22/min, plateau pressure (Pplateau) <30 mmHg. Static compliance (SC) and dynamic compliance (DC) were calculated according to the following formulas: SC = Vt/Pplateau - PEEP DC = Vt/Ppeak - PEEP

The IAP was measured in each patient according to Cheatham and Safcsak's technique [8], but instead of using a transducer, a column with a scale in centimeter of water (cmH_2_O) was added to the urinary drainage system (Figure 1). Two measurements at end expiration with a 6-h interval were done during the first 24 h by the same investigator in order to avoid interobserver variability. The intravesical saline volume was 100 ml as was a common practice at that time. With the patient in supine position, the zero reference was placed at the mid-axillary line using the superior iliac crest as the reference point. Each IAP value was obtained by manometry (cmH_2_O) and recalculated in millimeter mercury using the conversion factor (1 cmH_2_O = 0.74 mmHg). The two IAP values obtained in each patient were averaged, and the results were entered in a database. The total number of measurements was 200. As stated by the consensus, IAH was considered when the measured IAP values exceeded 12 mmHg [1].

Four ventilation modes were used: controlled mechanical ventilation (CMV), assisted/controlled ventilation (ACV), pressure support ventilation (PSV), and continuous positive airway pressure (CPAP). The patients were sedated to obtain a Ramsay score of 4 to 5 when receiving mechanical controlled ventilation and a Ramsay score of 2 to 3 in the ACV mode and PSV. Patients on CPAP ventilation were sedated only if necessary. No relaxing agents were used.

### Statistical analysis

The Statistical Package for Social Sciences (SPSS for Windows version 16.0 software, SPSS Inc., Chicago, IL, USA) was used in order to organize, validate, and analyze the collected data. Indicators of central tendency and dispersion: medians, means, standard deviations, and 95% confidence intervals (CI) were estimated for quantitative variables, while frequencies and percentages were used for qualitative variables. Two-sample paired '*t*' test was used to evaluate the differences of means in two samples, and the Mann-Whitney *U *test was used to evaluate the differences of means in two independent samples and non-normality assumption. The Kruskal-Wallis test was used to compare more than two means. The chi-square test with Yates' correction for continuity or Fisher's exact test was used wherever appropriate in order to identify the differences between categorical variables. Pearson correlation test was also applied to find out any association between ventilation parameters and IAP.

A multiple linear regression model was applied for the whole patient population to assess the independent influence of age, gender, body mass index (BMI), and MV on IAP. With the ventilated group, a multiple linear regression model was applied for assessing the independent influence of age, gender, BMI, PEEP, minute volume, and PSV mode on IAP.

A *p *value of <0.05 was considered to be significant for all the statistical tests. Tables and figures were constructed in order to present the most relevant findings. The primary endpoint in the study was the effect of MV on IAP values.

The protocol was approved by the local ethics committees, and informed consent was provided by patients or next of kin before the study inclusion. The IAP measurements had no interference with other diagnostic or therapeutic procedures, according to the Council for International Organizations of Medical Sciences recommendations [9] and Helsinki Declaration [10].

## Results

One hundred patients were included in this study. The general characteristics are shown in Table 1. Ventilated and non-ventilated groups were similar in quantity, gender, age, and BMI. Concerning APACHE II, there was a trend to higher values in ventilated patients, though there was no statistical significance.

**Table 1:** Characteristics of the critically ill patients in the mechanically ventilated and non-ventilated groups

	Total (*n *= 100)	MV (*n *= 50)	Non-MV (*n *= 50)	*p *value
Female (*n*)	50	24 (48%)	26 (52%)	0.841^a^
Age (years)	46.6 ± 17.0	45.9 ± 15.5	47.3 ± 18.6	0.666^b^
BMI (kg/m^2^)	24.3 ± 3.5	24.2 ± 3,5	24.3 ± 3.6	0.893^b^
APACHE II	8.5 ± 4.7	9.2 ± 5.4	7.8 ± 3.8	0.125^b^
IAP	5.1 ± 3.7	6.7 ± 4.1	3.5 ± 2.4	* <0.0001^b^*
APP	87.7 ± 17.6	85.3 ± 21.7	90.1 ± 11.9	0.173^b^
IAH	6	6 (12.0)	0 (0.0%)	*0.027^c^*
Medical	53 (53.0)	26 (52.0%)	27 (54%)	1.000
Surgical	47 (47.0)	24 (48.0%)	23 (46.0%)	
Elective	31 (66.0%)	18 (72.0%)	13 (59.1%)	^d^
Emergency	5 (10.6%)	2 (8.0%)	3 (13.6%)	
Trauma	11 (23.4%)	5 (20.0%)	6 (27.3%)	
ICU stay	5.6 ± 5.5	6.5 ± 6.5	4.6 ± 4.3	0.087^b^
ICU mortality	21 (21.0%)	18 (36.0%)	3 (6.0%)	* <0.0001^c^*

^a^Chi-square test with Yates' correction; ^b^two independent sample *t *tests; ^c^Fisher's exact test; ^d^*p *value was not calculated as the expected frequency was <5 in more than 25% of the cells. MV, mechanical ventilation; BMI, body mass index; APACHE, Acute Physiology and Chronic Health Evaluation; IAP, intra-abdominal pressure; APP, abdominal perfusion pressure; IAH, intra-abdominal hypertension; ICU, intensive care unit.

The IAP was significantly higher (*p* < 0.0001) in the ventilated patients (6.7 ± 4.1 mmHg) compared to the non-ventilated patients (3.5 ± 2.4 mmHg), and concordantly, the abdominal perfusion pressure (APP, calculated as mean arterial pressure minus IAP) was lower in the ventilated patients (85.3 ± 21.7 mmHg). IAH was present in only six ventilated patients (12% in the MV group and 6% for the whole group). As can be seen, the proportions of medical and surgical patients were similar. The majority (66%) of the surgical patients was elective (31/47).

The ICU stay was not significantly longer in the ventilated group compared to the non-ventilated patients (*p *= 0.087). ICU mortality was higher in ventilated patients compared to the non-ventilated ones (*p* < 0.0001).

Table 2 shows the information in relation to the presence or absence of IAH. Both groups were similar concerning age, gender, BMI, and the other variables, and only the IAP was notably different between the two groups (*p* < 0.0001).

**Table 2:** Characteristics of IAH and non-IAH critically ill patients

	Total (*n *= 50	IAH (*n *= 6)	Non-IAH (*n *= 44)	*p *value
Female (*n*)	24 (48.0)	1 (16.7)	23 (52.3)	0.192^a^
Age (years)	45.9 ± 15.5	51.7 ± 7.6	45.1 ± 16.1	0.289^b^
BMI (kg/m^2^)	24.2 ± 3.5	26.5 ± 4.4	23.9 ± 3.2	0.199^b^
APACHE II	9.2 ± 5.4	9.7 ± 4.9	9.2 ± 5.5	0.753^b^
IAP	6.7 ± 4.1	14.9 ± 3.2	5.6 ± 2.8	* <0.000^b^*
APP	85.3 ± 21.7	70.3 ± 23.1	87.3 ± 21.0	0.078^b^
MV	50 (100)	6	44	
Medical	26 (52.0%)	3 (50.0%)	23 (52.3%)	1.000^a^
Surgical	24 (48.0%)	3 (50.0%)	21 (47.7%)	
Elective	18 (36.0%)	3 (50.0%)	15 (34.1%)	^c^
Emergency	2 (4.0%)	0 (0.0%)	2 (4.5%	
Trauma	5 (10.0%)	0 (0.0%)	5 (11.4%)	
ICU stay	6.5 ± 6.5	5.8 ± 5.2	6.6 ± 6.7	0.940^b^
ICU mortality	32 (64.0%)	4 (66.7%)	28 (63.6%)	1.000^a^

^a^Fisher's exact test; ^b^Mann-Whitney *U *test; ^c^*p *value was not calculated as the expected frequency was <5 in more than 25% of the cells. IAH, intra-abdominal hypertension; BMI, body mass index; APACHE, Acute Physiology and Chronic Health Evaluation; IAP, intra-abdominal pressure; APP, abdominal perfusion pressure; MV, mechanical ventilation; ICU, intensive care unit.

**Figure 2:**
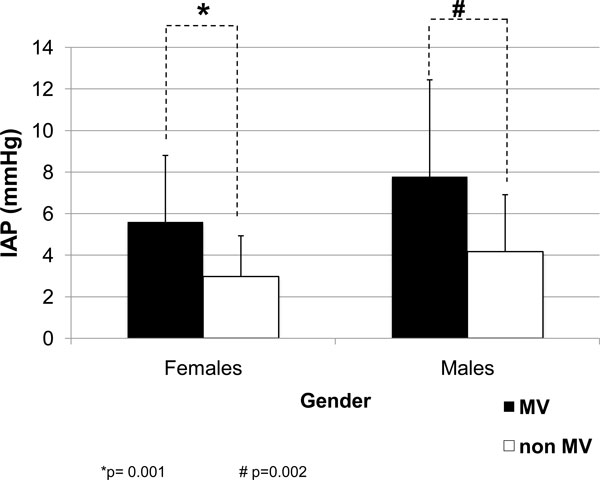
**IAP according to gender in the ventilated and non-ventilated critically ill patients**. Two independent sample *t *tests: *p *= 0.001 (asterisk) and *p *= 0.002 (hash sign). MV, mechanical ventilation; IAP, intra-abdominal pressure.

**Figure 3:**
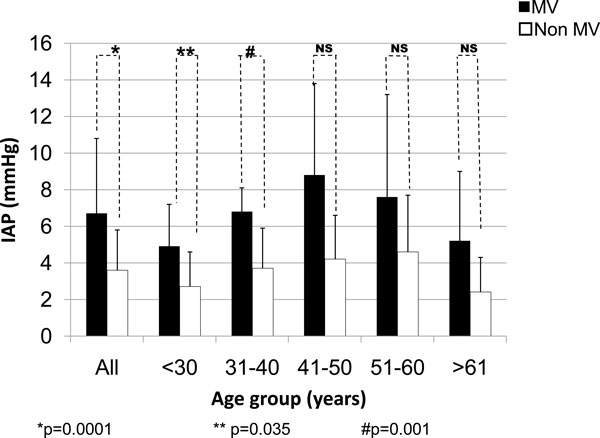
**IAP in the ventilated and non-ventilated patients according to the age groups**. Two independent sample *t *tests: *p *= 0.0001 (asterisk), *p *= 0.035 (double asterisk), and *p *= 0.001 (hash sign). MV, mechanical ventilation; IAP, intra-abdominal pressure; NS, no significance.

According to gender, the IAP values were higher in males regardless of the use of MV or not. The IAP values in ventilated patients were always higher (Figure 2). With regard to the age, the IAP values were always higher in ventilated patients (Figure 3) with a strong statistical difference, especially in the three age groups of patients under 50 years.

**Figure 4:**
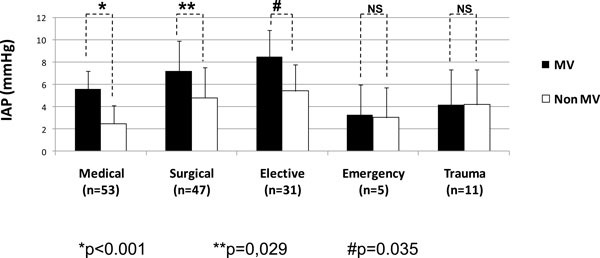
**IAP according to the diagnostic category in the ventilated and non-ventilated critically ill patients**. Mann-Whitney *U *test: *p* < 0.001 (asterisk), *p *= 0.029 (double asterisk), and *p *= 0.035 (hash sign). MV, mechanical ventilation; IAP, intra-abdominal pressure; NS, no significance.

According to the major diagnostic categories (Figure 4), the IAP values were also significantly higher in medical patients (*p* < 0.001) as well as in surgical ones (*p *= 0.029) undergoing MV. Among the surgical patients, the elective patients had the most important differences.

**Figure 5:**
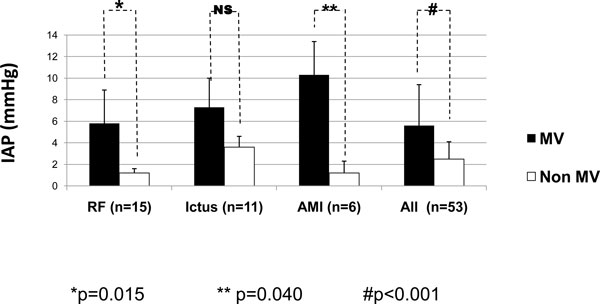
**IAP according to the diagnostic category in the ventilated and non-ventilated medical patients**. Mann-Whitney *U *test: *p *= 0.015 (asterisk), *p *= 0.040 (double asterisk), and *p* < 0.001 (hash sign). MV, mechanical ventilation; IAP, intra-abdominal pressure; RF, respiratory failure; AMI, acute myocardial infarction; NS, no significance.

**Figure 6:**
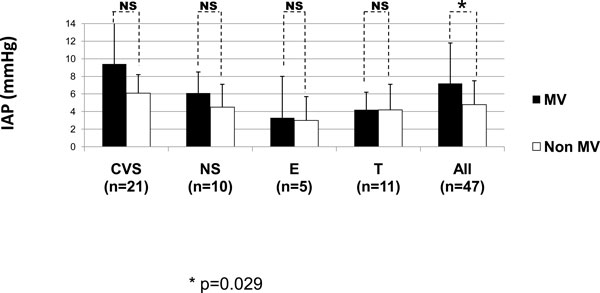
**IAP according to the diagnostic category in the ventilated and non-ventilated surgical patients**. The elective patients are represented by CVS and NS patients. Mann-Whitney U test: *p *= 0.029 (asterisk). MV, mechanical ventilation; IAP, intra-abdominal pressure; CVS, cardiovascular surgery; NS, neurosurgery; E, emergency; T, trauma; NS, no significance.

**Figure 7:**
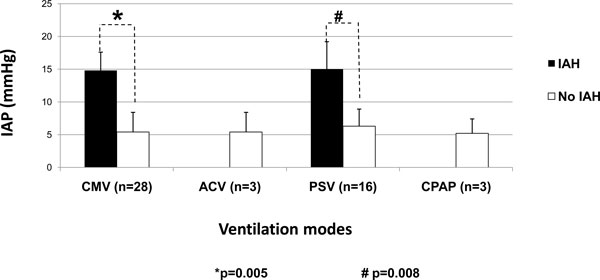
**IAP in the ventilated patients according to the different ventilation modes**. Mann-Whitney *U *test: *p *= 0.005 (asterisk) and *p *= 0.008 (hash sign). IAH, intra-abdominal hypertension; IAP, intra-abdominal pressure; CMV, controlled mechanical ventilation; ACV, assisted/controlled ventilation; PSV, pressure control ventilation; CPAP, continuous positive airway pressure.

According to the indications for MV, some of the subgroups influenced the IAP values, as can be seen in Figures 5 and 6. In Figure 5 concerning the medical diagnostics, some diagnoses (CHF, MD, CSN sepsis, and 'others') were skipped as the comparison between the mean values was not possible as there was only one patient in one of the groups. Patients with AMI and RF had the most significant differences (*p *= 0.015 and *p *= 0.04, respectively). Figure 6 shows that the IAP values in the surgical patients were higher in patients on MV compared to non-ventilated patients though the differences were not significant. Patients with IAH were only seen on CMV and PSV (Figure 7).

In general, ventilation parameters were similar through the different ventilation modes (Table 3), but as shown in Table 4, there was a trend to higher PEEP values in the IAH patients' group. In addition, as can be seen in Table 5, there was a positive correlation between PEEP and IAP (*p *= 0.018).

**Table 3:** Ventilatory parameters and ventilation mode

Parameter	Total (*n *= 50)	CMV (*n *= 28)	PSV (*n *= 16)	ACV (*n *= 3)	CPAP (*n *= 3)	*p*^a^
RR (breath/min)	17.5 ± 4.5	16.9 ± 5.3	18.0 ± 3.1	20 ± 3.8	18.3 ± 4.2	0.399
Vmin (l/min)	9.4 ± 2.7	9.3 ± 3	9.7 ± 2	9.0 ± 1.8	9.2 ± 4.9	0.503
Vt (ml/kg)	8.1 ± 2.2	8.0 ±1.7	8.8 ± 3.1	7.1 ± 1.1	6.7 ± 0.8	0.116
Ppeak (cmH_2_O)	24.0 ± 11.7	24.4 ± 11.7	25.5 ± 13.2	20.6 ± 4.7	15.6 ± 6.6	0.108
Pplateau (cmH_2_O)	13.1 ± 8.7	14.3 ± 10.5	12.0 ± 5.8	13.0 ± 6.2	7.6 ± 0.5	0.123
PEEP (cmH_2_O)	2.1 ± 2.5	2.2 ± 2.4	2.0 ± 2.7	2.7 ± 2.5	1.3 ± 2.3	0.417
SC (ml/cmH_2_O)	79.3 ± 57.8	79.1 ± 59.3	79.3 ± 59.4	75.3 ± 77.6	85.4 ± 41.6	0.548
DC (ml/cmH_2_O)	32.0 ± 21.6	32.7 ± 24.7	30.4 ± 18.0	27.0 ± 8.9	39.3 ± 26.6	0.548

^a^Mann-Whitney *U *test. CMV, controlled mechanical ventilation; PSV, pressure support ventilation; ACV, assisted/controlled ventilation; CPAP, continuous positive airway pressure; RR, respiratory rate; Vmin, minute volume; Vt, tidal volume; Ppeak, peak pressure; Pplateau, plateau pressure; PEEP, positive end-expiratory pressure; SC, static compliance; DC, dynamic compliance.

**Table 4:** Ventilatory parameters according to IAH definition

Parameter	Total	IAH	Non-IAH	*p *value^a^
RR (breath/min)	17.6 ± 4.6	18.7 ± 3.2	17.4 ± 4.7	0.402
Vmin (l/min)	9.4 ± 2.8	10.1 ± 2.0	9.3 ± 2.8	0.243
Vt (ml/kg)	8.2 ± 2.3	8.2 ± 3.3	8.2 ± 2.2	0.540
Ppeak (cmH_2_O)	24.1 ± 11.7	20.7 ± 10.0	24.5 ± 12.0	0.370
Pplateau (cmH_2_O)	13.1 ± 8.8	12.5 ± 8.3	13.2 ± 8.9	0.858
PEEP (cmH_2_O)	2.1 ± 2.5	3.7 ± 2.5	1.9 ± 2.4	0.080
SC (ml/cmH_2_O)	79.4 ± 73.7	110.4 ± 84.6	75.1 ± 53.1	0.325
DC (ml/cmH_2_O)	32 ± 21.6	41.3 ± 24.4	30.7 ± 21.2	0.282

^a^Mann-Whitney *U *test. IAH, intra-abdominal hypertension; RR, respiratory rate; Vmin, minute volume; Vt, tidal volume; Ppeak, peak pressure; Pplateau, plateau pressure; PEEP, positive end-expiratory pressure; SC, static compliance; DC, dynamic compliance.

**Table 5:** Correlations for IAP vs. ventilatory parameters

Variable	Correlation	Ppeak	Pplateau	PEEP	RR	Vt	SC	DC
IAP	Pearson correlation	-0.139	-0.170	0.333	-0.029	0.754	0.232	0.638
	*p *value	0.334	0.239	**0.018**	0.957	0.084	0.658	0.173

ventilator parameters (*n *= 50)

IAP, intra-abdominal pressure; RR, respiratory rate; Vt, tidal volume; Ppeak, peak pressure; Pplateau, plateau pressure; PEEP, positive end-expiratory pressure; SC, static compliance; DC, dynamic compliance.

In a multiple linear regression analysis for the total population of critically ill patients (adjusting for age, gender, BMI, and MV), male gender (*p* < 0.05), BMI (*p* < 0.05), and MV (<0.001) were independently related with IAP, but not the age (*p *= 0.495) (Table 6). Multiple linear regression analysis for the ventilated patients (adjusting for age, gender, BMI, PEEP, minute volume, and ventilation mode) shows that the male gender (*p* < 0.05), PEEP (*p* < 0.05), and PSV (<0.001) were independently related to IAP, but not the age (*p *= 0.740), BMI (*p *= 0.061) and minute volume (*p *= 0.950) (Table 7). In relation to ICU outcome, the non-survivors had only higher APACHE II scores (*p *= 0.022) (Table 8).

**Table 6:** Results of the multiple linear regression for the critically ill patients (*n *= 100)

Variable	Coefficient *b*^a^	Coefficient *β*^b^	CI 95%		*p*
			**IL**	**SL**	
Constant	-2.093		-6.737	2.550	0.373
Age	-0.014	-0.063	-0.054	0.026	0.475
Males	1.575	0.212	0.281	2.870	**0.018**
BMI	0.228	0.213	0.33	0.422	**0.022**
MV	3.129	0.421	1.838	4.421	** <0.0001**
Model summary	*R*^2 ^= 0.276; adjusted *R*^2 ^= 0.246				

^a^Unstandardized coefficient; ^b^standardized coefficient. BMI, body mass index; MV, mechanical ventilation; IL, inferior limit; SL, superior limit.

**Table 7:** Results of the multiple linear regression for the ventilated critically ill patients (*n *= 50)

Variable	Coefficient *b*^a^	Coefficient *β*^b^	CI 95%		*p*
			**IL**	**SL**	
Constant	-4.764		-13.451	3.922	0.275
Age	0.012	0.045	-0.061	0.085	0.740
Males	2.808	0.342	0.536	5.081	**0.017**
BMI	0.300	0.250	-0.014	0.614	0.061
Vmin	0.013	0.009	-0.400	0.426	0.950
PEEP	0.556	0.331	0.127	0.985	**0.012**
PSV^c^	2.896	0.329	0.485	5.307	**0.020**
Model summary	*R*^2 ^= 0.322; adjusted *R*^2 ^= 0.227				

^a^Unstandardized coefficient; ^b^standardized coefficient; ^c^reference category: CMV. BMI, body mass index; Vmin, minute volume; PEEP, positive end-expiratory pressure; PSV, pressure support ventilation; CMV, controlled mechanical ventilation; IL, inferior limit; SL, superior limit.

**Table 8:** Characteristics of the critically ill patients according to ICU outcome

	Total (*n *= 100)	Survivors (*n *= 79)	Non-survivors (*n *= 21)	*p *value
Female (*n*)	50 (50.0%)	42 (53.2%)	8 (16%)	0.326^a^
Age (years)	46.6 ± 17.0	46.1 ± 17.2	48.2 ± 16.4	0.537^b^
BMI (kg/m^2^)	24.3 ± 3.5	24.3 ± 3.7	24.08 ± 2.7	0.787^b^
APACHE II	8.5 ± 4.7	7.9 ± 4.2	10.9 ± 5.7	**0.022^b^**
IAP	5.1 ± 3.7	5.2 ± 3.7	4.8 ± 3.9	0.651**^b^**
APP	87.7 ± 17.6	88.5 ± 14.1	84.4 ± 27.4	0.488^b^
IAH	6 (6.0%)	2 (9.5%)	4 (5.1%)	0.603^c^
Medical	53 (53.0%)	38 (48.1%)	15 (71.4%)	0.097^a^
Surgical	47 (47.0%)	41 (51.9%)	6 (28.6%)	
Elective	31	30 (38.0%)	1 (4.8%)	^d^
Emergency	5	5 (6.3%)	0 (0.0)	
Trauma	11	6 (7.6%)	5 (23.8%)	
ICU stay	5.6 ± 5.5	5.4 ± 5.7	6.4 ± 5.0	0.283^b^

^a^Chi-square test with Yates' correction; ^b^Mann-Whitney *U *test; ^c^Fisher's exact test; ^d^*p *value was not calculated as the expected frequency was <5 in more than 25% of the cells. BMI, body mass index; APACHE, Acute Physiology and Chronic Health Evaluation; IAP, intra-abdominal pressure; APP, abdominal perfusion pressure; IAH, intra-abdominal hypertension; ICU, intensive care unit.

## Discussion

In this study population, we observed that IAP was affected by MV, leading to higher IAP values, and this difference was also observed throughout the subsequent analysis performed in relation to gender, age, and diagnostic category. Moreover, the multiple linear regression showed that MV was an independent and predicting factor for the development of IAH in this cohort of critically ill patients. Our results support previous suggestions that artificial ventilation can exert a direct influence on the IAP due to increased intrathoracic pressures that are then transmitted to the abdomen [1,2,7].

In relation to gender, males had the higher IAP values, and this difference was observed also in the ventilated patients. This is an interesting observation that has only been reported in few studies previously. Sugerman et al. [11] found significant differences between IAP in male and female patients. Male patients had higher IAP values, and this was related to the sagittal diameter and the metabolic syndrome. Sánchez also reported a similar tendency [12], although the differences were not so important. According to this investigator, the central distribution of fat in males and the peripheral distribution in females can result in differences in the abdominal wall compliance (man having lower compliance). The influence of previous pregnancy states on the other hand can lead to increased abdominal wall compliance and, thus, lower IAP values in women. Finally, in our study, male gender was a predisposing and independent predictor for IAH in patients under MV.

The BMI was also independently associated with IAH. This relation has been reported previously in non-critically ill patients by Sanchez et al. [12] and Noblett [13], and in critically ill patients by Soler [14]. In addition, BMI was identified by Malbrain et al. as an independent factor for IAH in a multicenter study [15]. More recently, Soler et al. reported the influence of BMI on IAP in a cohort of 100 critically ill surgical patients regardless the position of the zero reference level during the measurement of IAP [16].

Among the ventilation parameters, only PEEP correlated significantly with IAP, albeit the fact that the PEEP levels observed were not so high in this study. Moreover, PEEP was the most important factor in relation to the development of IAH in the multiple regression analysis (for the whole population and in the group of patients on MV).

A review of the literature shows different results with regard to the relation between PEEP and IAP. For example, Sussman, the first to study this relation, could not find any relation between PEEP and IAP [17], while other investigators more recently have found a mild increase in IAP when PEEP is applied in patients with a baseline IAP below 12 mmHg [18]. Verzilli et al. [19] found an increase of IAP with moderate PEEP due to the transmission of the intrathoracic pressure to the abdomen in 13 selected patients with acute lung injury or adult respiratory distress syndrome. High PEEP decreases splanchnic perfusion [1,5,20]. The reduction of splanchnic blood flow is however limited at PEEP levels below 10 cmH_2_O but is more pronounced when PEEP levels are raised to 15 to 20 cmH_2_O. As Verzilli et al. stated the effects of PEEP on IAP values were such that they would increase the IAH grading, especially in patients with hypovolemia or high baseline IAP [19], suggesting that high PEEP levels may be a risk factor for IAH in selected ALI/ARDS patients.

Although in the ventilated patients we could not find any statistical differences with regard to the influence of the mode of ventilation on IAP, there were no IAH patients included in the ACV and CPAP modes. The multiple linear regression analysis showed that PSV was strongly associated with IAH. Although higher intrathoracic pressures can be observed or expected in controlled MV, in comparison to other ventilation modes, the use of sedation could result in improved static compliance hence lowering the IAP. While putting patients on PSV, ACV and CPAP could result in higher IAP values in view of the absence of sedation. We speculate that some unknown factors could have influenced our results. Maybe the random distribution of the patients and the small sample size of patients in ACV and CPAP modes can also explain these differences.

Some factors like body position, zero reference position, or interobserver variability did not influence our results [20-22] as these factors were controlled for. The technique for the IAP measurement was standardized, and all the measurements were done with the patients in supine position, with the zero reference at the mid-axillary line (iliac crest), and by the same investigator. A bit surprisingly, IAP was not associated with mortality in this population. As reported before in two multicenter studies, the mean IAP on admission is not considered an independent risk for mortality in this setting [15,23]. Only APACHE II was associated to mortality, confirming its usefulness as a predicting score.

This study has some limitations. First, although the measurement technique was standardized and applied by the same person to avoid interobserver variability, the amount of instilled saline into the bladder was 100 ml, higher than the recommended, and this could have resulted in overestimation of the IAP values. At the time when the study was conducted, it was common practice to use 100 to 200 ml of saline as priming solution. Nowadays, it is well known that large instillation volumes may cause overestimation of IAP as demonstrated by several investigators [1,24-27].

Second, a measurement bias is possible because all values were obtained in centimeters of water and recalculated to be expressed in millimeter mercury. Third, the selected casemix had of course an impact on the results. Our study is clearly different from previously performed studies since patients with abdominal surgical problems and those who received fluid overload (resulting in poor abdominal wall compliance) were excluded. As a result, the measurement of IAP was not used to evaluate the risk of IAH. That explains the relatively low prevalence of IAH in contrast with the previous reports [2,23,28-30] where the patients at risk for IAH were not excluded. In our opinion, this exclusion permitted a better focus on MV as a predisposing factor for IAH. Fourth, it would have given extra value to the results if IAP values could have been obtained before and after the initiation of MV.

## Conclusions

To our knowledge, this is the first multicenter observational study looking at the effect of MV on baseline IAP values in critically ill patients with no other apparent risk factors for IAH. Our results support the consensus definition statement [8] with regard to the influence of MV as a predisposing factor for the development of IAH: the use of MV was an independent predisposing factor for the development of IAH in this cohort of critically ill patients.

This study also confirms that PEEP is a predisposing factor for the development of IAH in a selected group of ventilated patients. No matter the cause, IAH is always deleterious and should be diagnosed and treated in time. Consequently, IAP should be followed-up carefully in the critically ill population, even when they have no other apparent risk of IAH, and especially if MV is applied.

## List of abbreviations used

ACV: assisted/controlled ventilation; AMI: acute myocardial infarction; APACHE: Acute Physiology and Chronic Health Evaluation; BMI: body mass index; CHF: congestive heart failure; CI: confidence interval; CMV: controlled mechanical ventilation; CPAP: continuous positive airway pressure: CVS: cardiovascular surgery; DC: dynamic compliance; E: emergency; IAH: intra-abdominal hypertension; IAP: intra-abdominal pressure; ICU: intensive care unit; MD: metabolic disorders; MV: mechanical ventilation; NS: neurosurgery; PEEP: positive end-expiratory pressure; Ppeak: peak pressure; Pplateau: plateau pressure; PSV: pressure support ventilation; RF: respiratory failure; RR: respiratory rate; SC: static compliance; T: trauma; Vmin: minute volume; Vt: tidal volume.

## Competing interests

The authors declare that they have no competing interests.

## Authors' contributions

CSM was involved in the design, collection, analysis, and interpretation of data and drafted the manuscript. TTB revised meticulously the database and made a new statistical analysis. TTB also collaborated in the interpretation of data and finally made a critical revision of the manuscript. All authors read and approved the final manuscript.
